# A New Ridge-Type Estimator for the Gamma Regression Model

**DOI:** 10.1155/2021/5545356

**Published:** 2021-06-18

**Authors:** Adewale F. Lukman, Issam Dawoud, B. M. Golam Kibria, Zakariya Y. Algamal, Benedicta Aladeitan

**Affiliations:** ^1^Department of Physical Sciences, Landmark University, Omu-Aran, Nigeria; ^2^Department of Biostatistics and Epidemiology, University of Medical Sciences, Ondo, Nigeria; ^3^Department of Mathematics, Al-Aqsa University, Gaza, State of Palestine; ^4^Department of Mathematics and Statistics, Florida International University, Miami, FL 33199, USA; ^5^Department of Statistics and Informatics, University of Mosul, Mosul, Iraq

## Abstract

The known linear regression model (LRM) is used mostly for modelling the QSAR relationship between the response variable (biological activity) and one or more physiochemical or structural properties which serve as the explanatory variables mainly when the distribution of the response variable is normal. The gamma regression model is employed often for a skewed dependent variable. The parameters in both models are estimated using the maximum likelihood estimator (MLE). However, the MLE becomes unstable in the presence of multicollinearity for both models. In this study, we propose a new estimator and suggest some biasing parameters to estimate the regression parameter for the gamma regression model when there is multicollinearity. A simulation study and a real-life application were performed for evaluating the estimators' performance via the mean squared error criterion. The results from simulation and the real-life application revealed that the proposed gamma estimator produced lower MSE values than other considered estimators.

## 1. Introduction

The gamma regression model (GRM) is generally adopted to model a skewed response variable that follows a gamma distribution with one or more independent variables. It is used in modelling the real-life data problems of several fields such as the medical sciences, health care economic, and automobile insurance claim [1]. When the positively skewed response variable follows a gamma distribution with a given set of independent variables, then it is preferred to use the gamma regression model [2–4]. As in linear regression models, the explanatory variables independence assumption rarely holds in practice, so the multicollinearity problem exists in the gamma regression models which means the maximum likelihood estimator (MLE) is unstable and gives high variances [5]. Consequently, constructing confidence intervals or testing the regression parameters of the model becomes difficult [6]. A lot of authors proposed different estimators for handling multicollinearity. The ridge estimator given by Hoerl and Kennard [7] is an alternative to MLE to overcome the multicollinearity in the linear regression model. The estimator has been extended to the generalized linear models (GLM) (see [8, 9]). Also, Månsson and Shukur [10] and Månsson [11] introduced the ridge estimator to the Poisson regression model and the negative binomial regression model, respectively. Kurtoglu and Ozkale [12] extend the Liu estimator of Liu [13] to the gamma regression model. Batah et al. [14] proposed a modified Jackknife ridge estimator by combining the ideas of the generalized ridge estimator and Jackknifed ridge estimator. Also, Algamal [3] developed the modified Jackknifed ridge gamma regression estimator. Recently, the modified version of the ridge regression estimator with two biasing parameters was proposed for both the LRM and GRM [15, 16]. Kibria and Lukman [17] proposed a new estimator called the ridge-type estimator and applied to the popular linear regression model.

The main objective portrayed in this article is to extend the new ridge-type estimator of Kibria and Lukman [17] to the GRM. The article organization is as follows: in Section 1, we proposed the new ridge-type gamma estimator, and then we derived its properties. Also, we have done the theoretical comparisons and have explained the estimation of the biasing parameter in Section 2. A simulation study is conducted to investigate and compare the performance of the new gamma estimator and some existing estimators in Section 3. We also analyzed a real-life data in Section 4. Finally, we have provided some concluding remarks in Section 5.

## 2. The Statistical Methodology

Consider the response variable *y*_*i*_ which follows the known gamma distribution with the parameter of the nonnegative shape *a*and the parameter of the nonnegative scale *b* with probability density function:

(1) $$\begin{array}{c} f\left(y_{i}\right)=\frac{y_{i}^{a-1}e^{-\left(y_{i}/b\right)}}{\Gamma \left(a\right)b^{a}},\;y_{i}\geq 0, \end{array}$$

where *E*(*y*_*i*_) = *ab* = *θ*_*i*_ and Var(*y*_*i*_) = *ab*^2^ = (*θ*_*i*_^2^/*a*),  *θ*_*i*_ = *e*^*x*_*i*_′*β*^. The log-likelihood function of (1) is

(2) $$\begin{array}{c} l\left(\beta \right)=\sum_{i=1}^{n}\left[\left(a-1\right)\ln\left(y_{i}\right)-\frac{y_{i}}{b}-a\;\ln\left(b\right)-\ln\left(\Gamma \left(a\right)\right)\right]. \end{array}$$

Equation (2) is solved iteratively since it is nonlinear in *β*using the Fisher scoring method as follows:

(3) $$\begin{array}{c} \beta^{t+1}=\beta^{t}+I^{-1}\left(\beta^{t}\right)S\left(\beta^{t}\right), \end{array}$$

where *t* is the iteration degree, *S*(*β*) = ∂*l*(*β*)/∂*β* and *I*^−1^(*β*) = (−*E*(∂^2^*l*(*β*)/∂*β*  ∂*β*′))^−1^. The last step for the estimated coefficients is considered as

(4) $$\begin{array}{c} {\hat{\beta }}_{\text{MLE}}=D^{-1}X^{\prime }\hat{Wz}, \end{array}$$

where $D=X^{\prime }\hat{W}X$, $\hat{W}=\text{diag}\left({\hat{\theta }}_{i}^{2}\right)$ matrix, ${\hat{\theta }}_{i}=\exp\left(x_{i}^{\prime }{\hat{\beta }}_{\text{MLE}}\right)$, and $\hat{z}$ is called the vector in *i*th element, $\hat{z}={\hat{\theta }}_{i}+\left(y_{i}-{\hat{\theta }}_{i}/{\hat{\theta }}_{i}^{2}\right)$. $\hat{W}$ and $\hat{z}$ are obtained by procedure of the Fisher scoring iterative (see [12, 18]). The matrix form of the covariance, the matrix of the mean squared error (MMSE), as well as the mean square error (MSE) are obtained by Algamal and Asar [19] and written, respectively, as follows:

(5) $$\begin{array}{c} \text{MMSE}\left({\hat{\beta }}_{\text{MLE}}\right)=\text{Cov}\left({\hat{\beta }}_{\text{MLE}}\right)=\phi D^{-1}, \end{array}$$

where *ϕ* = (1/*n* − *p*)∑_*i*=1_^*n*^((*y*_*i*_ − *θ*_*i*_)^2^/*θ*_*i*_^2^).

(6) $$\begin{array}{c} \text{MSE}\left({\hat{\beta }}_{\text{MLE}}\right)=\text{tr}\left(\text{MMSE}\left({\hat{\beta }}_{\text{MLE}}\right)\right)=\phi \sum_{j=1}^{p}\frac{1}{\gamma_{j}}, \end{array}$$

where *γ*_*j*_ is considered as an *j*th eigenvalue of the given matrix $D=X^{\prime }\;\hat{W}X$ and the notation *X*′is the transpose of *X*.

The gamma ridge estimator (GRE) is considered as

(7) $$\begin{array}{c} {\hat{\beta }}_{\text{GRE}}=D_{k}^{-1}{\hat{\beta }}_{\text{MLE}},\;k>0, \end{array}$$

where *D*_*k*_ = (Ι + *kD*^−1^) and *k* is the biasing parameter. The MMSE and MSE of GRE are given by

(8) $$\begin{array}{c} \text{MMSE}\left({\hat{\beta }}_{\text{GRE}}\right)=\text{Cov}\left({\hat{\beta }}_{\text{GRE}}\right)+\text{Bias}\left({\hat{\beta }}_{\text{GRE}}\right)\text{Bias}\left({\hat{\beta }}_{\text{GRE}}\right),\\ =\phi D_{k}^{-1}D^{-1}D_{k}^{-1}+k^{2}D_{k}^{-1}D^{-1}\beta \beta^{\prime }D^{-1^{\prime }}D_{k}^{-1^{\prime }}\\ \text{MSE}\left({\hat{\beta }}_{\text{GRE}}\right)=\text{tr}\left(\text{MMSE}\left({\hat{\beta }}_{\text{GRE}}\right)\right),\\ =\phi \overset{p}{\underset{j=1}{\sum }}\frac{\gamma_{j}}{{\left(\gamma_{j}+k\right)}^{2}}+k^{2}\overset{p}{\underset{j=1}{\sum }}\frac{\alpha_{j}^{2}}{{\left(\gamma_{j}+k\right)}^{2}}, \end{array}$$

where *α* = *P*′*β* such that *P* is the matrix of eigenvectors of *D*.

The gamma Liu estimator (GLE) is given by

(9) $$\begin{array}{c} {\hat{\beta }}_{\text{GLE}}={F_{d}\hat{\beta }}_{\text{MLE}},\;0<d<1, \end{array}$$

where *F*_*d*_ = (*D* + Ι)^−1^(*D* + *d*Ι) and *d* is the biasing parameter.

The MMSE and MSE of GLE are given by

(10) $$\begin{array}{c} \text{MMSE}\left({\hat{\beta }}_{\text{GLE}}\right)=\text{Cov}\left({\hat{\beta }}_{\text{GLE}}\right)+\text{Bias}\left({\hat{\beta }}_{\text{GLE}}\right)\text{Bias}{\left({\hat{\beta }}_{\text{GLE}}\right)}^{\prime }\\ =\phi F_{d}D^{-1}F_{d}+{\left(1-d\right)}^{2}{\left(D+Ι\right)}^{-1}\beta \beta^{\prime }{\left(D+Ι\right)}^{-1},\\ \text{MSE}\left({\hat{\beta }}_{\text{GLE}}\right)=\text{tr}\left(\text{MMSE}\left({\hat{\beta }}_{\text{GLE}}\right)\right)\\ =\phi \overset{p}{\underset{j=1}{\sum }}\frac{{\left(\gamma_{j}+d\right)}^{2}}{\gamma_{j}{\left(\gamma_{j}+1\right)}^{2}}+{\left(1-d\right)}^{2}\overset{p}{\underset{j=1}{\sum }}\frac{\alpha_{j}^{2}}{{\left(\gamma_{j}+1\right)}^{2}}. \end{array}$$

### 2.1. The New Gamma Estimator

For the known linear regression model, Kibria and Lukman [17] proposed the following new ridge-type estimator and called as the Kibria–Lukman (KL) estimator, which is defined as

(11) $$\begin{array}{c} {\hat{\beta }}_{\text{KL}}=W\left(k\right)M\left(k\right){\hat{\beta }}_{\text{OLS}},\;k>0, \end{array}$$

where *W*(*k*) = (Ι + *k*(*X*′*X*)^−1^)^−1^, *M*(*k*) = (Ι − *k*(*X*′*X*)^−1^), and ${\hat{\beta }}_{\text{OLS}}={\left(X^{\prime }X\right)}^{-1}X^{\prime }Y$.

In this study, we extend the KL estimator to the GRM and referred to the estimator as gamma KL estimator (GKL) which is written as follows:

(12) $$\begin{array}{c} {\hat{\beta }}_{\text{GKL}}=D_{k}^{-1}R_{k}{\hat{\beta }}_{\text{MLE}}, \end{array}$$

where *R*_*k*_ = (Ι − *kD*^−1^).

The bias and covariance matrix form of GKL estimator are gotten respectively as:

(13) $$\begin{array}{c} \text{Bias}\left({\hat{\beta }}_{\text{GKL}}\right)=\left(D_{k}^{-1}R_{k}-Ι\right)\beta , \end{array}$$

where $E\left({\hat{\beta }}_{\text{MLE}}\right)=\beta$ and

(14) $$\begin{array}{c} \text{Cov}\left({\hat{\beta }}_{\text{GKL}}\right)=\phi D_{k}^{-1}R_{k}D^{-1}R_{k}^{\prime }D_{k}^{-1^{\prime }}. \end{array}$$

So, the MMSE and MSE in terms of eigenvalues are defined, respectively, as

(15) $$\begin{array}{c} \text{MMSE}\left({\hat{\beta }}_{\text{GKL}}\right)=\text{Cov}\left({\hat{\beta }}_{\text{GKL}}\right)+\text{Bias}\left({\hat{\beta }}_{\text{GKL}}\right)\text{Bias}{\left({\hat{\beta }}_{\text{GKL}}\right)}^{\prime }\\ =\phi D_{k}^{-1}R_{k}D^{-1}R_{k}^{\prime }D_{k}^{-1^{\prime }}+\left(D_{k}^{-1}R_{k}-Ι\right)\beta \beta^{\prime }{\left(D_{k}^{-1}R_{k}-Ι\right)}^{\prime },\\ \text{MSE}\left({\hat{\beta }}_{\text{GKL}}\right)=\text{tr}\left(\text{MMSE}\left({\hat{\beta }}_{\text{GKL}}\right)\right)\\ =\phi \overset{p}{\underset{j=1}{\sum }}\frac{{\left(\gamma_{j}-k\right)}^{2}}{\gamma_{j}{\left(\gamma_{j}+k\right)}^{2}}+4k^{2}\overset{p}{\underset{j=1}{\sum }}\frac{\alpha_{j}^{2}}{{\left(\gamma_{j}+k\right)}^{2}}. \end{array}$$

### 2.2. The Theoretical Comparison for the Estimators

Some needed lemmas are stated as follows for comparing the estimators in theoretical.

**Lemma 1 .**

Suppose *n* × *n* matrices *F* is positive definite (p.d.) as well as *A* is p.d. (or *A*is nonnegative); then, *F* > *A* iff *λ*_max_(*AF*^−1^) < 1, where *λ*_max_(*AF*^−1^) is the max eigenvalue for the matrix *AF*^−1^[20].

**Lemma 2 .**

Suppose *R* is an *n* × *n* matrix which is p.d. and *α*be a vector; then, *R* − *αα*′is p.d. iff *α*′*R*^−1^*α* < 1 [21].

**Lemma 3 .**

Suppose that *α*_*i*_ = *L*_*i*_*y*, *i* = 1,2 be the given two linear estimators of *α*. Also, suppose $I=\text{Cov}\left({\hat{\alpha }}_{1}\right)-Cov\left({\hat{\alpha }}_{2}\right)$ is p.d., where $\text{Cov}\left({\hat{\alpha }}_{i}\right)i=1,2$ is considered as the covariance matrix form of ${\hat{\alpha }}_{i}$ and $b_{i}=\text{Bias}\left({\hat{\alpha }}_{i}\right)=\left(L_{i}X-I\right)\alpha$, *i* = 1,2. Consequently,

(16) $$\begin{array}{c} \Delta \left({\hat{\alpha }}_{1}-{\hat{\alpha }}_{2}\right)=\text{MMSE}\left({\hat{\alpha }}_{1}\right)-\text{MMSE}\left({\hat{\alpha }}_{2}\right)=\sigma^{2}I+b_{1}b_{1}^{\prime }-b_{2}b_{2}^{\prime }\text{ is p.d}., \end{array}$$

if *b*_2_′[*σ*^2^*I* + *b*_1_′*b*_1_]*b*_2_ < 1, where $\text{MMSE}\left({\hat{\alpha }}_{i}\right)=\text{Cov}\left({\hat{\alpha }}_{i}\right)+b_{i}b_{i}^{\prime }$ [22].

#### 2.2.1. Comparison of GKL and MLE

**Theorem 1 .**

${\hat{\beta }}_{\text{GKL}}$ is better than ${\hat{\beta }}_{\text{MLE}}$ if

(17) $$\begin{array}{c} \beta^{\prime }{\left(D_{k}^{-1}R_{k}-Ι\right)}^{\prime }\left[\phi \left(D^{-1}-D_{k}^{-1}R_{k}D^{-1}R_{k}^{\prime }D_{k}^{-1^{\prime }}\right)\right]\left(D_{k}^{-1}R_{k}-Ι\right)\beta <1. \end{array}$$

**Proof**

The difference of the dispersion is

(18) $$\begin{array}{c} \text{Cov}\left({\hat{\beta }}_{\text{MLE}}\right)-\text{Cov}\left({\hat{\beta }}_{\text{GKL}}\right)=\phi \left(D^{-1}-D_{k}^{-1}R_{k}D^{-1}R_{k}^{\prime }D_{k}^{-1^{\prime }}\right). \end{array}$$

We observed that *D*^−1^ − *D*_*k*_^−1^*R*_*k*_*D*^−1^*R*_*k*_′*D*_*k*_^−1′^ is positive definite (p.d.) since (*γ*_*j*_ + *k*)^2^ − (*γ*_*j*_ − *k*)^2^ = 4*γ*_*j*_*k* > 0, for *k* > 0. By Lemma 3, the proof is done.

#### 2.2.2. Comparison of GKL and GRE

**Theorem 2 .**

${\hat{\beta }}_{\text{GKL}}$ is superior to ${\hat{\beta }}_{\text{GRE}}$ if

(19) $$\begin{array}{c} \beta^{\prime }{\left(D_{k}^{-1}R_{k}-Ι\right)}^{\prime }\left[V_{1}+\left(D_{k}^{-1}-Ι\right)\beta \beta^{\prime }\left(D_{k}^{-1}-Ι\right)\right]\left(D_{k}^{-1}R_{k}-Ι\right)\beta ,\\ \lambda_{\max}\left(AF^{-1}\right)<1, \end{array}$$

where

(20) $$\begin{array}{c} V_{1}=\phi \left(D_{k}^{-1}D^{-1}D_{k}^{-1^{\prime }}-D_{k}^{-1}R_{k}D^{-1}R_{k}^{\prime }D_{k}^{-1}\right),\\ A=kD_{k}^{-1}D^{-1}D_{k}^{-1},\\ F=2D_{k}^{-1}D_{k}^{-1}. \end{array}$$

**Proof**

(21) $$\begin{array}{c} V_{1}=\phi \left(D_{k}^{-1}D^{-1}D_{k}^{-1^{\prime }}-D_{k}^{-1}R_{k}D^{-1}R_{k}^{\prime }D_{k}^{-1}\right)\\ =\phi \left(D_{k}^{-1}D^{-1}D_{k}^{-1^{\prime }}-D_{k}^{-1}\left(Ι-kD^{-1}\right)D^{-1}\left(Ι-kD^{-1}\right)D_{k}^{-1}\right)\\ =\phi kD^{-1}\left(F-A\right)D^{-1}, \end{array}$$

where *A* = *kD*_*k*_^−1^*D*^−1^*D*_*k*_^−1^ and *F* = 2*D*_*k*_^−1^*D*_*k*_^−1^.

Clearly, for the biasing parameters *k* > 0 and 0 < *d* < 1, *F* > 0 as well as *A* > 0. *F* − *A* > 0 if *λ*_max_(*AF*^−1^) < 1, where *λ*_max_(*AF*^−1^) is the max eigenvalue of the matrix form *AF*^−1^. By Lemma 1, the proof is done.

#### 2.2.3. Comparison of GKL and GLE

**Theorem 3 .**

${\hat{\beta }}_{\text{GKL}}$ is superior to ${\hat{\beta }}_{\text{GLE}}$ if

(22) $$\begin{array}{c} \beta^{\prime }{\left(D_{k}^{-1}R_{k}-Ι\right)}^{\prime }\left[V_{2}+{\left(1-d\right)}^{2}{\left(D+Ι\right)}^{-1}\beta \beta^{\prime }{\left(D+Ι\right)}^{-1}\right]\left(D_{k}^{-1}R_{k}-Ι\right)\beta \leq 1, \end{array}$$

where *V*_2_ = *ϕ*(*F*_*d*_*D*^−1^*F*_*d*_′ − *D*_*k*_^−1^*R*_*k*_*D*^−1^*R*_*k*_′*D*_*k*_^−1′^).

**Proof**

The difference of the dispersion is

(23) $$\begin{array}{c} V_{2}=\phi \left(F_{d}D^{-1}F_{d}^{\prime }-D_{k}^{-1}R_{k}D^{-1}R_{k}^{\prime }D_{k}^{-1^{\prime }}\right). \end{array}$$

We observed that *F*_*d*_*D*^−1^*F*_*d*_′ − *D*_*k*_^−1^*R*_*k*_*D*^−1^*R*_*k*_′*D*_*k*_^−1′^ is p.d. since (*γ*_*j*_ + *k*)^2^(*γ*_*j*_ + *d*)^2^ − (*γ*_*j*_ − *k*)^2^(*γ*_*j*_ + 1)^2^ > 0 for *k* > 0 and 0 < *d* < 1. By Lemma 3, the proof is done.

#### 2.2.4. Estimation of Parameter *k*

The optimal value of *k* in ${\hat{\beta }}_{\text{GKL}}$ is adopted from the KL estimator of the study of Kibria and Lukman [17] as follows:

(24) $$\begin{array}{c} k=\frac{\phi }{\left(2\beta_{j}^{2}+\left(\phi /\gamma_{j}\right)\right)}. \end{array}$$

The optimal value of *k* given in (24) depends on the unknown parameters *ϕ* and *β*_*j*_^2^. Therefore, we put the corresponding unbiased estimators instead of them. Consequently,

(25) $$\begin{array}{c} \hat{k}=\frac{\hat{\phi }}{\left(2{\hat{\beta }}_{\text{MLE}\left(j\right)}^{2}+\left(\hat{\phi }/\gamma_{j}\right)\right)}. \end{array}$$

## 3. Simulation Design

R 3.4.1 programming language is adopted for the simulation design of this study. Following Algamal [19], the response variable is generated as follows:

(26) $$\begin{array}{c} y_{i}∼G\left(\frac{\theta^{2}}{\upsilon },\;\frac{\upsilon }{\theta }\right), \end{array}$$

where *θ* = exp(*X*′*β*) and , *υ*denotes *θ*^2^. The parameter vector, *β*, is chosen such that ∑_*j*=1_^*p*^*β*_*j*_^2^ = 1[1, 23, 24]. Following Kibria [25] and Kibria and Banik [26], the given explanatory variables are obtained as follows:

(27) $$\begin{array}{c} x_{ij}={\left(1-\rho^{2}\right)}^{1/2}w_{ij}+\rho w_{ip+1},\;i=1,2,\ldots ,n;\;j=1,2,\ldots ,p,\;p+1, \end{array}$$

where *w*_*ij*_ are generated from standard normal and *ρ*^2^ is the correlation between the explanatory variables. The values of *ρ*in this study are chosen to be 0.95, 0.99, and 0.999. We obtained the mean function for *p* = 4 and 7 explanatory variables, respectively, for the following sample sizes: 20, 50, and 200. For each replicate, we compute the mean square error (MSE) of the estimators by using the following equation:

(28) $$\begin{array}{c} \text{MSE}\left(\beta_{i}^{*}\right)=\frac{1}{1000}\sum_{i=1}^{1000}{\left(\beta_{i}^{*}-\beta \right)}^{\prime }\left(\beta_{i}^{*}-\beta \right), \end{array}$$

where *β*_*i*_^*∗*^ would be any of the following estimators (MLE, GRE, GLE, and GLK). The smaller the mean square error value is, the better the estimator is. The biasing parameters for GRE and GLE are obtained as follows:

(29) $$\begin{array}{c} \hat{k}=\min{\left(\frac{\hat{\phi }}{{\hat{\beta }}_{\text{MLE}\left(j\right)}^{\prime 2}}\right)}_{j=1}^{p},\\ \hat{d}=\min{\left(0,\frac{{\hat{\beta }}_{\text{MLE}\left(j\right)}^{2}}{\left(\hat{\phi }/\gamma_{j}+{\hat{\beta }}_{\text{MLE}\left(j\right)}^{2}\right)}\right)}_{j=1}^{p}. \end{array}$$

We examined two shrinkage parameters for the proposed estimator. They are defined as follows:

(30) $$\begin{array}{c} {\hat{k}}_{1}=\min{\left(\frac{\hat{\phi }}{\left(2{\hat{\beta }}_{MLE\left(j\right)}^{2}+\left(\hat{\phi }/\gamma_{j}\right)\right)}\right)}_{j=1}^{p},\\ {\hat{k}}_{2}=\sqrt{{\hat{k}}_{1}}. \end{array}$$

**Figure 1:**
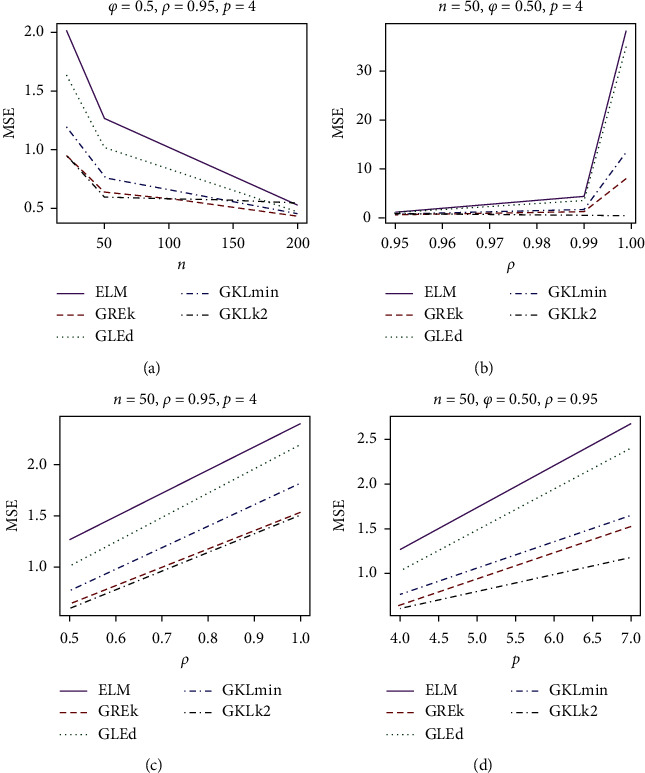
Estimated MSE vs. different values of (a) *n*, (b) *ρ*, (c) *φ*, and (d) *p*.

The simulation results for different values of *n*, *φ*, and *ρ* are presented in Tables 1 and 2 for *p* = 4 and 7, respectively. For a graphical representation, we also plotted MSE vs *n*, *ρ*, *φ*, and *p* in Figure 1.

It was observed from both Tables 1 and 2 and Figure 1 that the MSE increases as the level of multicollinearity increases keeping other variables constant. For instance, when *n* = 50, for the MLE, the MSE increases from 1.265 to 38.172 as the level of multicollinearity, *ρ*rises from 0.95 to 0.999 for given *ϕ* = 0.5 and *p* = 4. We also observed that, as the explanatory variables increases from *p* = 4 to *p* = 7, the MSE increases provided other variables are kept constant. For instance, when *n* = 20 for *ρ* = 0.99 and *ϕ* = 1, the MSE for the GRE-k rises from 6.753 to 19.071. Also, when other variables are fixed, increasing the sample size *n* results in a decrease in the MSE for all the estimators', for example, the MSE value of GLE-d for *n* = 200, *ϕ* = 0.5, * p* = 7, and *ρ* = 0.95 reduces from 1.282 to 1.549. Furthermore, the MSE increases as the dispersion parameter *ϕ* increases from 0.5 to 1. The maximum likelihood estimator performs least as expected because of the effect of multicollinearity on the estimator. The result in Tables 1 and 2 and Figure 1 shows that the GKL outperforms other estimators. Since the performance of the proposed estimator GKL depends on its biasing parameter, we examined two different biasing parameters for GKL estimator and observed that the GKL estimator performs best with the biasing parameter, ${\hat{k}}_{2}.$ The simulation result further supports the theoretical results that the performance of GKL estimator is the best. The performance of the GRE and GLE is better than that of the MLE. Furthermore, we explored the performance of the proposed estimator and the existing estimators by analyzing a real-life data in Section 4.

## 4. Real-Life Data: Algamal Data

The chemical dataset adopted in this study was employed in the study of Algamal [3, 19]. He employed the quantitative structure-activity relationship (QSAR) model to study the relationship between the biological activities *IC*_50_ of 65 imidazo [4, 5-b] pyridine derivatives – an anticancer compound – and 15 molecular descriptors. The QSAR model is widely used in the following fields: chemical sciences, biological sciences, and engineering. The linear regression model is popularly used to model the QSAR relationship between the response variable (biological activity) and one or more physiochemical or structural properties which serve as the explanatory variables especially when the response variable is normally distributed [27]. However, the regression modelling is employed when the response variable is skewed [3, 19, 24, 28]. In this study, following Algamal [3, 19], the variables of interest are described in Table 3.

According to Algamal [3, 19]; the response variable, *y*, follows a gamma distribution. Using the chi-square goodness of fit test, author examined that the response variable is well fitted to the gamma distribution with test statistic (*p* value) given as 9.3657 (0.07521). Algamal [19] reported that the correlation coefficient between the following variables, Mor21v and Mor21e, SpMax3_Bh(s) and ATS8v, SpMaxA_D and MW and finally MW and ATS8v, is greater than 0.9 and interpreted as high correlation. The eigenvalues of $X^{\prime }\;\hat{W}X$ are 7.6687E + 8, 1.3238E + 6, 85791, 5523.6, 358.71, 250.51, 148.46, 42.731, 27.239, 18.015, 9.1197, 8.6175, 5.7748, 2.4292, 1.6532, and 0.3659, respectively. Thus, the condition number, CN is computed as follows:

CN = $\sqrt{\max\left(\text{eigenvalue}\right)/\min\left(\text{eigenvalue}\right)}$ = 45777.7 which indicates the presence of severe multicollinearity [19]. The results of the gamma regression model and the mean square error are presented in Table 4.

The result in Table 4 agrees with the simulation results. The performance of the MLE is the worst in terms of possessing the highest MSE. The proposed estimator with the biasing parameter ${\hat{k}}_{2},$ in this order has the least mean square error followed by ${\hat{k}}_{\min}$, GRE-k and GLE-d estimators. Recall in the simulation study GKL with ${\hat{k}}_{2}$ as the shrinkage parameter performed the best.

## 5. Some Concluding Remarks

The Kibria–Lukman [17] estimator was developed to circumvent the problem of multicollinearity for the linear regression model. This estimator is in the class of the ridge regression and the Liu-type regression estimator, and it has a single biasing parameter. In gamma regression model, multicollinearity is also a threat for the performance of the maximum likelihood estimator (MLE) in the estimation of the regression coefficients. The gamma ridge (GRE) and the gamma Liu estimator (GLE) has been introduced in the previous study to mitigate the problem of multicollinearity. Since, Kibria and Lukman [17] claimed that the KL estimator outperforms the ridge and Liu estimator in the linear regression model, which motivated us to develop the gamma KL (GKL) estimator for the effective estimation in the GRM. We derived the statistical properties of GKL estimator and compared it theoretically with the MLE, GRE, and GLE. Furthermore, a simulation study and a chemical data analysis were conducted in support of the theoretical study. The simulation and application result show that GKLE with ${\hat{k}}_{2}$ as the shrinkage parameter performed the best. In conclusion, the use of the GKL estimator is preferred when multicollinearity exists in the known gamma regression model.

## Figures and Tables

**Table 1:** Estimated mean squared error when *p* = 4.

*ϕ*	*n*	*ρ*	MLE	GRE-*k*	GLE-*d*	GKL (${\hat{k}}_{\min}$)	GKL (${\hat{k}}_{2}$)
0.5	20	0.95	2.008	0.949	1.643	1.193	0.942
		0.99	8.195	2.761	7.156	4.083	2.018
		0.999	78.599	23.305	75.070	37.119	17.929
	50	0.95	1.265	0.643	1.025	0.763	0.601
		0.99	4.277	1.257	3.532	1.799	1.102
		0.999	38.172	8.044	35.320	13.298	7.051
	200	0.95	0.544	0.444	0.478	0.459	0.435
		0.99	0.923	0.467	0.682	0.551	0.463
		0.999	5.068	0.554	4.067	1.522	0.545

1	20	0.95	3.514	1.758	3.113	2.025	1.357
		0.99	15.677	6.753	14.558	8.226	4.568
		0.999	154.076	63.790	150.439	79.217	61.203
	50	0.95	2.671	1.528	2.406	1.655	1.155
		0.99	11.034	5.410	10.200	6.003	2.205
		0.999	105.109	48.863	102.240	54.610	26.562
	200	0.95	0.628	0.449	0.546	0.473	0.445
		0.99	1.392	0.504	1.050	0.683	0.463
		0.999	9.837	3.220	8.355	2.948	1.276

**Table 2:** Estimated mean squared error when *p* = 7.

*ϕ*	*n*	*ρ*	MLE	GRE-*k*	GLE-*d*	GKL (${\hat{k}}_{\min}$)	GKL (${\hat{k}}_{2}$)
0.5	20	0.95	4.049	2.193	3.473	2.784	2.165
		0.99	17.213	6.962	15.174	10.464	6.451
		0.999	172.420	63.921	164.530	102.441	55.631
	50	0.95	2.393	1.525	2.188	1.800	1.520
		0.99	7.742	3.192	7.036	4.588	2.509
		0.999	69.729	22.843	67.015	36.936	22.786
	200	0.95	1.375	1.155	1.282	1.252	1.103
		0.99	2.131	1.210	1.750	1.561	1.207
		0.999	9.941	1.658	8.325	4.507	1.431

1	20	0.95	7.397	4.424	6.884	5.075	3.476
		0.99	34.889	19.071	33.216	22.709	11.262
		0.999	356.808	192.852	350.583	231.657	123.844
	50	0.95	4.790	3.348	4.651	3.564	2.779
		0.99	19.784	12.398	19.291	13.428	5.905
		0.999	191.838	116.591	189.700	126.654	35.276
	200	0.95	1.644	1.462	1.549	1.402	1.348
		0.99	3.269	1.583	2.839	2.125	1.437
		0.999	20.402	4.716	18.550	9.311	4.049

**Table 3:** Description of the variable.

Variable names	Description
Mor21v	Signal 21/weighted by van der Waals volume
Mor21e	Signal 21/weighted by Sanderson electronegativity
IC3	Information content index
MW	Molecular weight
SpMaxA_D	Normalized leading eigenvalue from topological distance matrix
ATS8v	Broto–Moreau autocorrelation of lag 8 weighted by van der Waals volume
GATS4p	Geary autocorrelation of lag 4 weighted by polarizability
SpMax8_Bh(p)	Largest eigenvalue *n*. 8 of Burden matrix weighted by polarizability.
SpMax3_Bh(s)	Largest eigenvalue *n*. 3 of Burden matrix weighted by l-state.
P_VSA_e_3	P_VSA-like on Sanderson electronegativity, bin 3
TDB08m	3D topological distance-based descriptors; lag 8 weighted by mass
RDF100m	Radial distribution function: 100/weighted by mass
MATS7v	Moran autocorrelation of lag 7 weighted by van der Waals volume
MATS2s	Moran autocorrelation of lag 2 weighted by l-state
HATS6v	Leverage-weighted autocorrelation of lag 6/weighted by van der Waals volume

**Table 4:** Application result using the proposed and existing estimators.

Coef.	MLE	GRE-*k*	GLE-*d*	GKL (${\hat{k}}_{\min}$)	GKL (${\hat{k}}_{2},$)
tpecretnI	−0.1568	−0.1597	−0.1568	−0.1624	−0.1573
MW	0.0158	0.0155	0.0158	0.0155	0.0148
IC3	0.8251	0.8254	0.8251	0.8255	0.8260
SpMaxA_D	−0.4681	−0.4418	−0.4681	−0.4407	−0.3816
ATS8v	−2.3347	−2.3161	−2.3347	−2.3165	−2.2691
MATS7v	−1.1565	−1.1382	−1.1565	−1.1392	−1.0903
MATS2s	−2.2127	−2.1479	−2.2127	−2.1452	−1.9987
GATS4p	−2.7097	−2.6510	−2.7097	−2.6511	−2.5068
SpMax8_Bh(*p*)	2.8041	2.7426	2.8041	2.7425	2.5930
SpMax3_Bh(s)	0.4082	0.3994	0.4082	0.3991	0.3790
P_VSA_e_3	0.0016	0.0017	0.0016	0.0017	0.0020
TDB08m	−1.3127	−1.1859	−1.3127	−1.1811	−0.8954
RDF100m	−0.0004	−0.0004	−0.0004	−0.0005	−0.0006
Mor21v	−0.8682	−0.8448	−0.8682	−0.8446	−0.7882
Mor21e	−0.0504	−0.0593	−0.0504	−0.0597	−0.0795
HATS6v	−0.5290	−0.4030	−0.5290	−0.3803	−0.1723
d/*k*		0.0077	0.9999	0.0824	0.2871
MSE	5.5599	3.5062	5.5599	3.2351	1.6397

## Data Availability

The data used to support the findings of this study are available upon request.
